# URBAN ENVIRONMENT AND AFFECTIVE STATES IN REAL TIME: AN ECOLOGICAL MOMENTARY ASSESSMENT STUDY OF OLDER ADULTS

**DOI:** 10.1093/geroni/igac059.3085

**Published:** 2022-12-20

**Authors:** Peichao Liang, Yingqi Guo, On Fung Chan, Cheryl Hiu-Kwan Chui, Yuqi Liu, Shiyu Lu, Terry Lum

**Affiliations:** The University of Hong Kong, Hong Kong, Hong Kong; The Hong Kong Polytechnic University, Hong Kong, Hong Kong; The University of Hong Kong, Hong Kong, Hong Kong; The University of Hong Kong, Hong Kong, Hong Kong; South China University of Technology, Guangzhou, Guangdong, China (People’s Republic); City University of Hong Kong, Kowloon, Hong Kong; The University of Hong Kong, Hong Kong, Hong Kong

## Abstract

Older adults are more dependent on their surrounding environment. Extensive research has demonstrated beneficial effects of both nature and built environment on mental health of older people. However, most previous research used cross-sectional designs failed to test the intraindividual variability between environment, behavior, and mental wellness in daily life. We used ecological momentary assessment (EMA), activity sensors, and GPS tracking to examine the association between real-time environment, mobility and activity, and momentary affect among older adults in Hong Kong. Data collection and data processing was conducted from December 2021 to May 2022. 168 older adults aged 65 to 84 received seven EMA prompts per day during a fifteen-day period, and completed a total of 17,345 momentary assessments of affective states, mobility, and activities. A set of GPS-derived indicators were used to measure the real-time environment. To disaggregate the between- and within-person effects, we used multilevel models to estimate three dimensions of affect, i.e., valence, calmness, and energetic arousal, in EMA observations, nested within individual participants. Preliminary results indicate significant concurrent associations between environmental attributes and momentary affect at the within-person level, while the between-person differences appear to be either null or modest. Being out of home is associated with higher valence ratings (b=0.04, p=0.0427), while exposure to green is associated with a lower level of energetic arousal (b=-0.03, p=0.0163). Greater walkability is consistently associated with higher momentary affect ratings in three dimensions, but these associations are not statistically significant. Implications of these findings for promoting healthy aging will be discussed.

